# Effects of vasicine in neuroinflammatory zebrafish model

**DOI:** 10.6026/97320630019595

**Published:** 2023-05-31

**Authors:** Balasundaram Arthi, Darling Chellathai

**Affiliations:** 1 Department of Pharmacology, Sri Ramachandra Medical College and Research Institute, Sri Ramachandra Institute of Higher Education and Research, Porur, Chennai-600116, Tamil Nadu, India; 2 Department of Pharmacology, Sri Ramachandra Medical College and Research Institute, Sri Ramachandra Institute of Higher Education and Research, Porur, Chennai-600116, Tamil Nadu, India

**Keywords:** Neuroinflammation, Neurodegeneration, Zebrafish, PCR, gel electrophoresis

## Abstract

Chronic neuroinflammation produces cytotoxic effects and aggravates neurodegeneration. Cognitive impairment was considered an early symptom of many neurodegenerative diseases. Therefore, it is of interest to evaluate the in-vivo efficacy of vasicine for treating neuroinflammation-induced cognitive impairments in the zebrafish model. The Neurobehavioral activity was evaluated with a dive tank test, swim motion test, plus maze test, turn angle test, and color preference test in three neuroinflammatory zebrafish models. Gene expression analysis was done for neuroinflammatory markers (IL-10, IL-15, IL-13, NogoA, Fetuin-A, BDNF, NAA, CXCL2, Osteopontin) using polymerase chain reaction and gel electrophoresis technique. Behavioral parameters and biochemical evaluations revealed that vasicine was effective in treatment against neuroinflammatory models of surgery, chemical, xenotransplantation. Attenuation of cognitive dysfunction in all three neuroinflammation zebrafish models by vasicine in this study may recommend vasicine as a potential molecule for treating neuroinflammatory and neurodegenerative diseases.

## Background:

Zebrafish are promising as an interesting novel animal model for many neurodegenerative diseases, including neuroinflammatory and other demyelinating diseases [1-2 3]. The zebrafish central nervous system (CNS) is a simplified version of the mammalian nervous system and contains majority of cell types, connections and structures of the mammalian brain and spinal cord [4] Zebrafish mRNA is detectable from 2 days post fertilization by in situ hybridization and is detectable by electron microscopy from 3 days post fertilization [5, 6]. Most of the myelin and oligodendrocyte genes are highly conserved in the zebrafish; however, there are some differences. The major protein in zebrafish myelin is P0, rather than PLP [17, 8]. Though neuro-inflammation has a protective role, it has been described as a double-edged sword as it may also induce or aggravate neurodegeneration through cytotoxic effects, resulting in neuromotor impairments [9,10,11 12,13,14,15 16]. Neurodegenerative diseases featured both neuroinflammation and neurodegeneration through demyelination and neuroaxonal loss which may also be categorized under chronic immune mediated central nervous system (CNS) disorder. Here, differential diagnosis of neuroinflammation and neurodegeneration was difficult and not fully understood whether neurodegeneration is an independent process or neuroinflammation related. In this outlook, Koudriavtseva T & Mainero C (2016) have elaborated that neuroinflammation protects against neurodegeneration by stimulating regeneration in acute and limited CNS injury. And stated that, chronic neuroinflammation increases neurodegeneration and disrupts neuroprotection. This causes increase in detrimental neuroinflammation and weakens regeneration. This insufficient regeneration and, extent of neuroinflammation and neurodegeneration exist simultaneously in different degree based on different factors like localization, lesion pathology, and phase of disease and age of immune system [17]. Thus, these findings cleared the doubts for relating neuro-inflammation and neuro-degeneration. Cognitive impairment (CI) was considered as early symptom of many neurodegenerative diseases and, in severe state as a causal factor for dementia [18], which is fifth largest cause of death as stated by a systematic analysis for the Global Burden of Disease Study 2016 [19]. Despite many drugs available for treating CI, it is still evidenced that a large proportion of patients fail to regain premorbid state. Phosphodiesterase 7B1 (PDE7B1) was shown to have potential role in causing cognitive impairment and dementia [20, 21]. This may be a likely target for treatment of cognitive impairment in neuroinflammation. Quinazoline derivatives were shown to possess inhibitory activity to PDE7 enzymes [22]. Vasicine, a quinazoline alkaloid from Adhatoda Vasica [23] was screened for its protective effects against cognitive impairments in neuroinflammatory models. The aim of the present study is to evaluate in-vivo efficacy of vasicine for treating neuroinflammation induced cognitive impairments in zebrafish model.

##  Material and Methods:

## Animals:

Eight Groups of 27 adult fish each were housed at light dark cycle of 14/10. Temperature of 28 degree Celsius was maintained in 10 liter water tanks with continuous aeration and bio filtration. Good Animal Practice as per Institutional Animal Ethics Committee in accordance with Committee for the Purpose of Control and Supervision of Experiments (CPCSEA), India, was followed with adherence to established protocols. Zebrafish husbandry, the drug and food treatment protocols were followed as per previous studies [24]. Fish were housed together, separated to be fed individually and regrouped immediately at least 4 hours prior to assay. Fish fed with vehicle (standard Ringer's solution of 6.5g NaCl, 0.42g KCl, 0.25g CaCl2 and 0.2g of sodium bicarbonate per liter) mixed feed served as control Group. Dosing was done for 14 consecutive days. The Vasicine treated groups were compared with three models of neuroinflammation (surgery induced, chemical induced and xenotransplantation). Chemical method was developed and standardized in this study using metronidazole. Group allocation of fish was detailed (Table 1). Compounds were dosed orally by mixing with the fish feed pellets. Fish were fed, three pellets a day for 14 consecutive days prior to assay. Concentration of compounds dosed was controlled by the number of pellets containing the compound and the quantity of compound per pellet. Feed pellets without drug but with vehicle were used as placebo. Behavioral tests listed were performed as described in previous literature. Behavioral parameters included Dive tank test for anxiety, Swim motion for motor response, Maze test for memory and learning, Angle of turn for hypersensitivity and epileptic movement, Color preference for reward behavior. Biochemical tests and pathological examination were performed as described in previous literature after euthanizing the fish, as per the guidelines.

## Induction of neuro-inflammation in Zebrafish:

Surgical Induction: Brain was injured in the brain stem region. A 0.5mm incision at the mid ventral region of the medulla was made between the cerebellum and Medulla oblongata and just above the isthmic region [25].

## Induction through Xenotransplantation of inflamed neuronal cells:

Neuronal precursor cells were co-cultured with leukocytes and 10ul of 3X105 cells per ml were injected into the brain stem [26].

## Toxin Induced:

Metronidazole was fed orally at a concentration of 14ug/day for 14 days and fish were maintained under hypoxic environment to induce neuropathy [27].

## Euthanasia:

All euthanasia was performed during the light phase, in a procedure room adjacent to the fish housing facility. Then fish was placed into a holding tank containing slurry of approximately equal amounts of ice and water such that fish would be placed into water maintained at 0 to 4 °C yet would not directly contact the ice. Fish were transferred to the ice water bath. A camera was mounted above the euthanasia setup for recording to determine time to loss of righting reflex and monitor frequency of stressful behaviors. Zebrafish were placed into the iced water bath for 60 min [28].

## Behavioral Tests:

## Dive tank test:

Fish was place in a tank of length X height 30cmX30cm. They were allowed to acclimatize under light of 1400 lumens for 15 minutes and recordings were made. Measurements were made at a period of 12 hours after dosing. Time spent at the bottom of the tank and at the top of the tank was noted for 3 minutes [29].

## Swim motion Test:

Fish was place in a tank of length X height 30cmX30cm. They were allowed to acclimatize under light of 1400 lumens for 15 minutes and recordings were made. Measurements were made at a period of 12 hours after dosing. The observation tank was divided into four zones by drawing vertical lines at equal distances, at a length x breath of 7.5 cm per zone. The number of lines that adult zebrafish crossed for a period of 3 minutes was counted [30].

## Plus Maze test:

Fish was place in a tank of 30 cm which was divided into 4 sections of 7.5cms each. They were allowed to acclimatize under light of 1400 lumens for 15 minutes and recordings were made. Measurements were made at a period of 12 hours after dosing. Fish was placed in one end of the tank and a food pellet was dropped in the other end of the tank. Time taken for the fish to swim through the maze tank to reach the food pellet was recorded [31].

## Angle of Turn Test:

Fish was place in a tank of length X height 30cmX30cm. They were allowed to acclimatize under light of 1400 lumens for 15 minutes and recordings were made. Measurements were made at a period of 12 hours after dosing. Video was recorded of the fish swimming and video was analyzed by Kinovea software for angle of turn [32].

## Color Preference Test:

Fish was place in a tank of length X height 30cmX30cm. They were allowed to acclimatize under light of 1400 lumens for 15 minutes and recordings were made. Measurements were made at a period of 12 hours after dosing. One half of the tank was covered with red sheet and the other with a blue sheet. The time spent at the blue zone was recorded [33, 34,35,36, 37,38,39, 40].

## Biochemical Tests:

## Quantification of Biomarkers using gene expression method:

Total RNA was extracted from brain samples of zebrafish following manufacturer's instructions for the TRIzol RNA isolation kit. The brain tissue was homogenized individually in 500 µmicro;L TRIzol, and total RNA was DNase-treated with a TURBO DNA-Free Kit (Ambion). The RNA quality was determined with gel electrophoresis. Gene expressions previously shown to be related with neuroinflammation were evaluated with quantitative reverse transcription PCR (RT-PCR). Zebrafish genome was screened from the site ZFIN.com and reference RNA sequence was identified. The sequences were pasted in primer 3 Tool and primers were designed. Primer pairs (Table 2) were tested in an RT-PCR that was incubated at 95°C for 1 minute, followed by 40 cycles of 95°C for 1 minute, 55°C for 20 seconds, and 72°C for 45 seconds, then 72°C for 2 minutes. Products were then run out on a 1% gel to check for product amplification. Amplified reverse transcripts are added into the additional wells of the gel. The gel is run at 80-150 V until the dye line is approximately 75-80% of the way down the gel. The Gel is visualized under UV illuminator and quantified with ImageJ software. Expression levels are calculated in reference to control as arbitrary units. By adding the primer to the extracted RNA will allow the replication of DNA coding for neuroinflammation biomarker if the extracted RNA contains the biomarker. Here the primer is added to amplify the existing meagre RNA which may be present in the extracted ZF brain RNA of induced neuroinflammation models. Hence this study will confirm & validate whether the induced models in zebrafish are mimicking neuro-inflammation or not.[41,42, 43,44].

## Histopathological Examination:

Fish were anesthetized with 15-degree Celsius water and sacrificed by a cut between the brain and the spinal cord. Brain was dissected and portions of mid brain were removed with a pin and knife. The tissue is smeared on a glass slide and stained with Hematoxylin & Eosin for 2 minutes each followed by water washes. Slides were viewed at 45X magnification and the number of degenerated neurons was counted for three fields per smear. Degenerate neurons were characterized by loss of cell structure, either swollen or constricted cells, irregular shaped cell membrane, stain relatively lighter with high rate of cell lysis during smear preparation. Heart and Tail pathology was also studied with this staining as in method revealed by Menke et al. [45].

## Statistical Analysis:

Statistical analysis was done using Prism 5 software from GraphPad. The unpaired Student's t-test with a 95% confidence interval was employed. The means of two unmatched groups were compared with the assumption that the values followed a Gaussian distribution. For multiple comparison tests of groups, a one-way ANOVA test at an alpha = 0.05 (95% confidence interval) was done.

## Result

## Effects of Vasicine on Dive Tank Behavior of Zebrafish:

In the dive tank test, the time spent by zebrafish at the bottom of the tank has been recorded and analyzed. The zebrafish of G3, G5 and G7 groups significantly spent more time at the bottom of the tank compared to the zebrafish of G1 and G2 groups. The vasicine-treated zebrafish of G4, G6, and G8 have significantly differed by spending less time compared to zebrafish of G3, G5 and G7 groups from day1 to day14 (Table 3,Table 4& Table 5).

## Effects of Vasicine on Swim Motion Behavior of Zebrafish:

The swimming activity of zebrafish was recorded to analyze the motor activity. The motor activity of the Zebrafish in G3, G5 and G7 groups significantly decreased in comparison to the zebrafish of G1 and G2 groups. The treatment groups zebrafish (G4, G6 and G8) had exhibited improved motor activity compared to G3, G5 and G7 group Zebrafish from day1 to day 14 (except for G3 and G4 group zebrafish on day 1) (Table 3,Table 4, Table 5).

## Effects of vasicine on plus-maze behavior of Zebrafish:

The time taken by the zebrafish to reach the food pellet was recorded. The plus-maze behavior was significantly low in the zebrafish of the G3, G5 and G7 groups compared to the G1 group and G2 group zebrafish. The G4, G6, and G8 treatment groups zebrafish exhibited significant improvement in plus-maze behavior in comparison to G3, G5, and G7 group zebrafish from day1 to day14 (except for G3 and G4 group zebrafish on day 1) (Table 3,Table 4, Table 5).

## Effects of vasicine on turn angle and epileptic behavior of Zebrafish:

The mean turn angle was significantly reduced in the zebrafish of G3, G5 and G7 groups in comparison to the G1 and G2 group zebrafish. This effect was significantly attenuated in the zebra fish of treatment groups (G4, G6, and G8) from day1 to day14 (Table 3,Table 4, Table 5).

## Effects of Vasicine on Color Preference Test:

The time spent by the zebrafish in the blue zone was recorded. The mean time spent was significantly lower in the G3, G4, and G5 groups zebrafish compared to the G1 and G2 groups Zebrafish. From day 1 to day 14, the Zebrafish of the G4, G6, and G8 groups spent significantly more time in the blue zone than the zebrafish of the G3, G5, and G7 groups (Table 3 Table 4,Table 5).

## Gene Expression Analysis:

**Figure 1:**
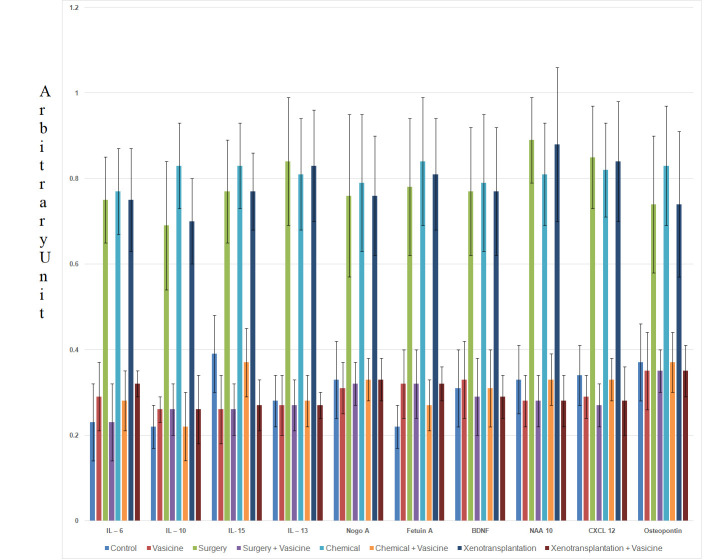
Gene expression of neuroinflammatory biomarkers in zebra fish. N=27. Control-control group fish, Vasicine-Vasicine group fish, Chemical-chemical induced group fish, Surgery-surgery induced group fish, Xenotransplantation-xenotransplantation induced group, Surgery+Vasicine-vasicine treated surgery induced group, Chemical + Vasicine- vasicine treated chemical induced group fish, Xenotransplantation+Vasicine-vasicine treated xenotransplantation group.

Gene expression of various inflammatory biomarkers was evaluated in this study. IL-6 gene expression was increased in surgery model, chemical model, and xenotransplantation model of neuroinflammation in comparison to control group. Vasicine treatment against neuroinflammation induced through surgery, chemical and xenotransplantation decreased the IL-6 expression in zebrafish. IL-10, IL-15, IL-13, NogoA, Fetuin A, BDNF, NAA, CXCL2, Osteopontin gene expression was increased in neuroinflammation induced models of surgery, chemical and xenotransplantation in comparison to control group. Vasicine treatment against neuroinflammation induced through surgery, chemical and xenotransplantation decreased the up-regulated expression of these genes (IL-10, IL-15, IL-13, NogoA, Fetuin A, BDNF, NAA, CXCL2, Osteopontin) in zebra fish (Figure 1).

**Figure 2:**
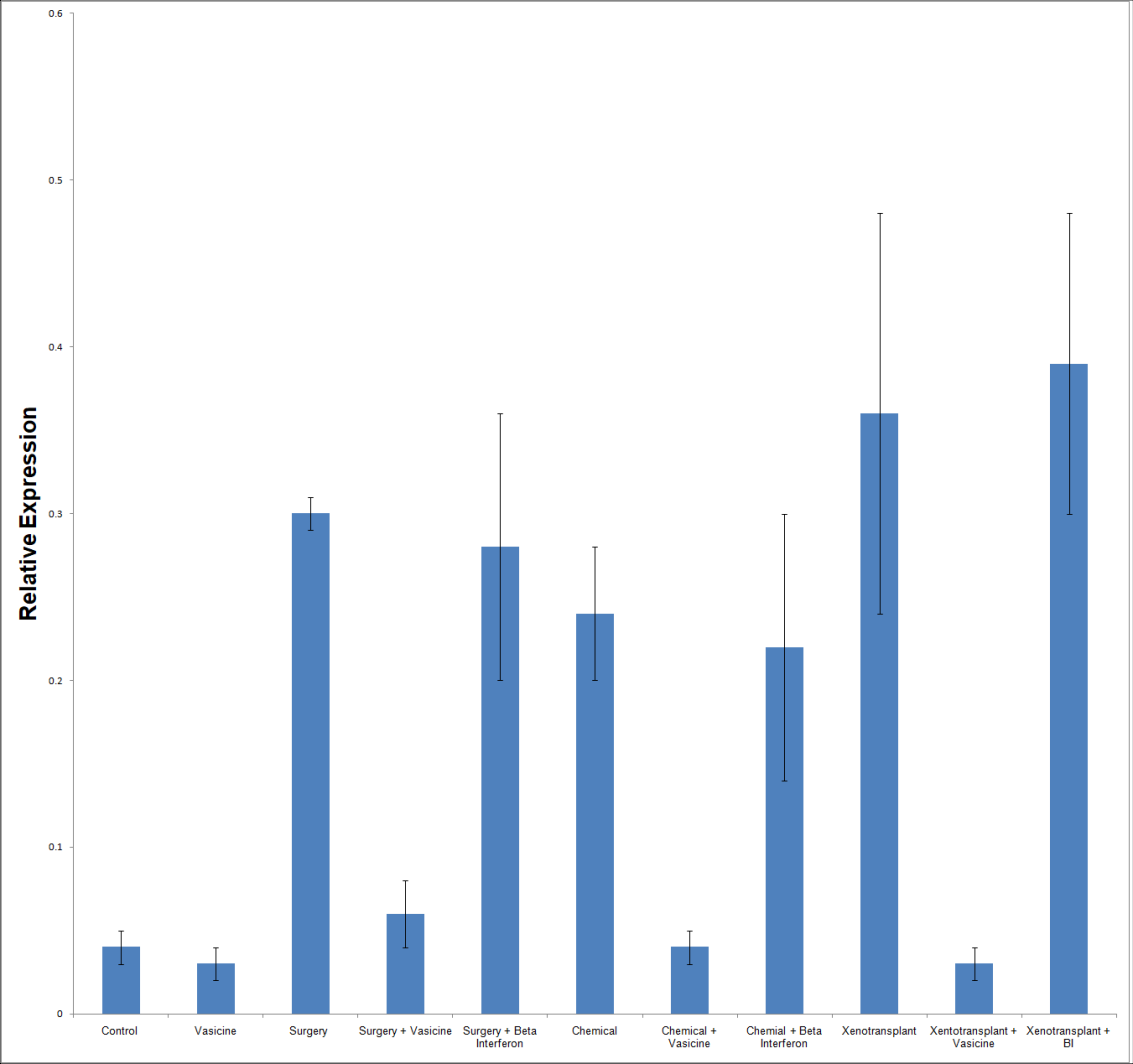
PDE7B Expression analysis in various models of study and Vasicine treated Zebra fish. N=27. Control-control group fish, Vasicine-Vasicine group fish, Chemical-chemical induced group fish, Surgery-surgery induced group fish, Xenotransplantation- xenotransplantation induced group, Surgery+Vasicine-vasicine treated surgery induced group, Chemical + Vasicine- vasicine treated chemical induced group fish, Xenotransplantation+Vasicine-vasicine treated xenotransplantation group, BI- Beta interferon.

Phosphodiesterase7B gene expression analysis revealed increased expression of PDE7B gene in inflammatory induced surgery, chemical and xenotransplantation models. Vasicine treatment against these neuroinflammatory models had attenuated the increased expression of PDE7B in zebrafish (Figure 2).

## Histopathological evaluation:

**Figure 3:**
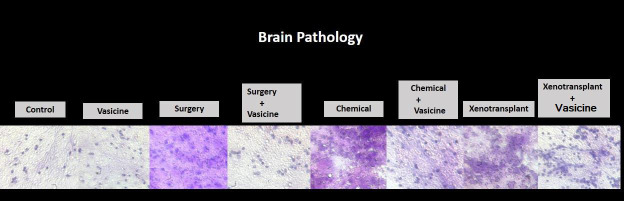
Histopathology of zebrafish brain in various models of neuroinflammation in the study. N=27. Control-control group fish, Vasicine-Vasicine group fish, Chemical-chemical induced group fish, Surgery-surgery induced group fish, Xenotransplantation- xenotransplantation induced group, Surgery+Vasicine-vasicine treated surgery induced group, Chemical + Vasicine- vasicine treated chemical induced group fish, Xenotransplantation+Vasicine-vasicine treated xenotransplantation group.

**Figure 4:**
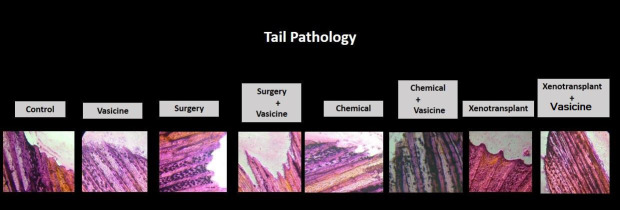
Tail Pathology of Zebrafish in various models of inflammation in the study. N=27. Control-control group fish, Vasicine- Vasicine group fish, Chemical-chemical induced group fish, Surgery-surgery induced group fish, Xenotransplantation- xenotransplantation induced group, Surgery+Vasicine-vasicine treated surgery induced group, Chemical + Vasicine- vasicine treated chemical induced group fish, Xenotransplantation+Vasicine-vasicine treated xenotransplantation group.

The H & E staining qualitative analysis revealed degenerated neurons in neuroinflammatory induced surgery, chemical and xenotransplantation models. Vasicine treatment against these neuroinflammatory models has attenuated the neuronal degeneration. Tail pathology showed variation in neuroinflammatory induced models both in texture and structure while vasicine treatment against these neuroinflammatory models showed normal structured tails resembling the control group fish tails (Figure 3, Figure 4).

## Discussion:

The aim of the present study is to evaluate the protective effects of vasicine against cognitive impairments in zebrafish neuroinflammatory models. The in-vivo efficacy study of vasicine against neuroinflammation model revealed protective effects of vasicine and improved cognitive impairments in this neuroinflammatory models. Dive tank test on 14th day of post induction of neuroinflammation showed that vasicine has improved the dive tank behavior (52.33 ± 0.58%, 65.33 ± 0.58%, 67.33 ± 0.58%) against all the three neuroinflammatory models surgery (75.67 ± 0.58%), chemical (94.67 ± 0.58%), and xenotransplantation (93%) respectively. Swim motion test on 14th day revealed that vasicine has improved motor activity (33.66 ± 0.58, 44.33 ± 0.58, 38.67 ± 0.58) against surgery (18.66 ± 0.58), chemical (23.67 ± 0.58) and xenotransplantation (22.67 ± 0.58) induced neuroinflammatory models. Plus maze test on the 14th day showed that vasicine has increased the %attention (84.67 ± 3.05, 88.33 ± 0.58, 73.67 ± 0.58) in comparison to all the three neuroinflammatory models surgery (44.67 ± 0.58), chemical (23.33 ± 0.58) and xenotransplantation (24%). Turn angle test on 14th day revealed that vasicine has improved the motor abilities (52.67 ± 0.58, 58.67 ± 0.58, 58.67 ± 0.58) against surgery (15), chemical (18.67 ± 0.58) and xenotransplantation (28.67 ± 0.58) induced neuroinflammation. Color preference test on 14th day revealed that vasicine has reduced the anxiety against neuroinflammatory models surgery, chemical, and xenotransplantation. Gene expression of various inflammatory biomarkers like IL-6, IL-13, IL-15, BDNF, NAA-10, Nogo A, Fetuin A, CXCL 12, and Osteopontin was increased in surgery model, chemical model, and xenotransplantation model of neuroinflammation in comparison to control group. Erta Maria et al. revealed that IL-6 expression increases in neuronal damage [46]. IL-13 was increased in neurodegenerative diseases [47]. Pan Weihong et al revealed increased production of IL-15 in neuroinflammatory diseases [48]. Lughetti Lorenzo et al revealed increased production of BDNF in neuroinflammation [49]. Pardon Marie Christine et al revealed increased levels of NAA in LPS induced neuroinflammation [50]. Nogo A was up regulated in cerebral inflammatory conditions [51]. Fetuin A was found to induce inflammation [52]. Vasicine treatment against neuroinflammation induced through surgery, chemical and xenotransplantation decreased the expression of IL-6, IL-13, IL-15, BDNF, NAA-10, Nogo A, Fetuin A, CXCL 12, and Osteopontin in zebrafish. This gene expression attenuation by vasicine can be correlated to the histological changes and finally to the improvement exhibited by vasicine treatment against the three neuroinflammatory models in the study.

A decline in the activities of antioxidases in ovalbumin and aluminum hydroxide administered animals and attainment of near normal in vasicine treated rats was demonstrated, concluding that lipid peroxidation and oxidative stress elicited by ovalbumin and aluminum hydroxide was considerably reduced with Vasicine treatment. This supports the present study which also shows reduction in lipid peroxidation activity by Vasicine [53]. Vasicine was shown to have a strong inhibitive activity against acetylcholinesterase (AChE) and butyrylcholinesterase (BChE), with IC50 values of 3.24 ± 0.08 µM and 0.10 ± 0.00 µM, respectively. It implied that Vasicine can be used for treatment of Alzheimer’s disease [54]. The present study shows beneficial activity of Vasicine in another neurodegenerative disease conditions and neuroinflammation. Wakhloo et al. 1980 showed that Vasicine hydrochloride was well tolerated by healthy human subjects (twenty-four) and is free from any undesirable side effects and has a wide safe dosage range [55]. Our study also revealed negligible toxicity of Vasicine, both in in-vitro and in-vivo study. A study reported that, 20 mg/kg body wt. of Vasicine given intramuscularly gets well absorbed thereby reaching a maximum concentration of about 50 µg/ml in blood in both pregnant and non-pregnant rats. Vasicine given intravenously in rat and mice showed high concentrations in uterus within 5 min and the peak was achieved after 10 min. In this case the half-life was 5 - 7 min, 1.5 and 2 h after intravenous, intramuscular, and subcutaneous administration respectively [56]. Similar results were obtained by Zutshi et al. (1980) in mice [57]. Very low concentration of Vasicine was observed after oral administration. Vasicine was metabolized in the liver to Vasicinone and other metabolites which contributes to the first pass effect and was found to be an important way of elimination of Vasicine [58]. Amla et al. (1987) had conducted a human trial in healthy volunteers to study the pharmacokinetics of Vasicine and reported that; 1.5mg/kg bolus i.v reached the peak in 15 min (65 ± 5µmol/L) and just after 4 hrs. 1/3 of the peak was detected [59]. Ram et al. (2007) determined the site of absorption of Vasicine in the intestine. They used everted sac method to assess the absorption. Duodenum was reported to have the maximum capacity to absorb isolated Vasicine from the methanolic and ethanolic extracts of Vasaka (82.3±5.3%) [60]. The improvement in behavioral response of zebrafish in vasicine treatment against neuroinflammation induced group fish may be related to attenuated inflammatory factors revealed through gene expression studies of IL-6, IL-13, IL-15, BDNF, NAA-10, Nogo A, Fetuin A, CXCL 12, Osteopontin.

## Conclusion:

Vasicine improved behavioral patterns in zebrafish neuroinflammatory models and was substantiated by changes in biochemical and histopathological evaluations. Vasicine has attenuated cognitive dysfunction in all three neuroinflammation zebrafish models. Through this study vasicine may be considered as a molecule for further research in neuroinflammatory and neurodegenerative diseases.

## Figures and Tables

**Table 1:** Study groups

**Group No**	**Group**	**Treatment**	**N**
Group 1	Control	Vehicle treated	27
Group 2	Vasicine	Extract control (Vasicine treated)	27
Group 3	Surgery Induced	Disease control 1(surgery)	27
Group 4	Vasicine treated +Surgery	Vasicine treated surgical neuroinflammation models	27
Group 5	Chemical Induced	Disease control 2(chemical)	27
Group 6	Vasicine treated +Chemical	Vasicine treated chemical induced neuroinflammation models	27
Group 7	Xenotransplantation Induced	Disease control 3(xenotransplantation)	27
Group 8	Vasicine treated + Xenotransplantation	Vasicine treated xenotransplantation neuroinflammation models	27
N=number of animals included

**Table 2:** Primer sequences

**S.No**	**Gene**	**Forward and Reverse Primer**	**Reference**
1	β- actin	f:5'cgagcaggagatgggaacc-3'	[34]
		r:5'caacggaaacgctcattgc-3'	
2	IL - 6	tttgcttacagaagattcaag	[35]
		atatggacaaacacaaactgt	
3	IL - 10	atttgtggagggctttcctt	[36]
		agagctgttggcagaatggt	
4	IL- 15	gctgcaatttttgcactgaa	[37]
		agcggtgctattctgatgct	
5	IL - 13	tcgggttttacgttgaaagg	[38]
		atctcctcctcagcctgaca	
6	Nogo A	atgcagccgcaggagtacat	[39]
		ggctgccgggtcacgact	
7	Fetuin A	agcggtgaaacgctacaact	[40]
		tgatccttcccacttccatc	
8	BDNF	caggacagcataggggaaaa	[41]
		ttgttttctcacgggtctcc	
9	NAA 10	gttctggcaaaaatggagga	[42]
		agtgaagagcagcccgatta	
10	CXCL 12	cacagtcccacagagaagca	[43]
		gttgatggcgttcttcaggt	
11	Osteopontin	ggaccaggcagctacagaag	[44]
		gaggagttggactcgctgtc	

**Table 3:** Surgery induced neuroinflammation model

**SURGERY MODEL**	**Day**	**Control**	**Vasicine**	**Surgery**	**Surgery + Vasicine** **P Value**
		Mean±SD	Mean±SD	Mean±SD	Mean±SD	
DIVE TANK TEST	1	49.67±0.58	49.67±0.58	72.33±0.58	69.67±0.58	0.001*,
	7	50.33±0.58	51.33±0.58	78.33±0.58	57.67±0.58	0.001*,
	14	49.33±0.58	50.67±0.58	75.67±0.58	52.33±0.58	0.0001#, 0.001*
SWIM MOTION TEST	1	43.33±0.58	45.67±0.58	13.67±0.58	13.67±0.58	1*,
	7	45.33±0.58	51.33±0.58	13.33±0.58	24.33±0.58	0.001*,
	14	49.33±0.58	50.67±0.58	18.67±0.58	33.67±0.58	0.0001#, 0.001*
PLUS MAZE TEST	1	99.33±0.58	100	36.67±0.58	36	0.001*,
	7	100	98.67±0.58	42.33±0.58	68.33±0.58	0.001*,
	14	99.67±0.58	100	44.67±0.58	84.67±3.05	0.0001#, 0.001*,
TURN ANGLE TEST	1	61.67±0.58	68.33±0.58	17.33±0.58	18.33±0.58	0.001*,
	7	62.33±0.58	63	13.66±0.58	26.33±0.58	0.001*,
	14	63.67±0.58	65	15	52.67±0.58	0.0001#, 0.001*,
COLOR PREFERENCE TEST	1	52.33±0.58	58.33±0.58	12.67±0.58	18.33±0.58	0.001*,
	7	52±0.58	56.67±0.58	23.67±0.58	33.67±0.58	0.001*,
	14	51.67±0.58	54.33±0.58	25	42.67±0.58	0.0001#, 0.001*,
N=27. Control-control group fish, Vasicine-vasicine group fish, Surgery-surgery induced group fish, and Surgery + Vasicine- vasicine treated surgery induced group fish. *-comparison between induction model and vasicine treated fish. #-comparison between day1 and day14 of vasicine treated inflammation model.

**Table 4:** Metronidazole induced neuroinflammation model

**CHEMICAL MODEL**	**Day**	**G1**	**G2**	**G5**	**G6**	**P Value**
		Mean±SD	Mean±SD	Mean±SD	Mean±SD	
DIVE TANK TEST	1	49.33±0.58	50.67±0.58	88.67±0.58	85.67±0.58	0.001*,
	7	49.33±0.58	50.67±0.58	93.67±0.58	77.33±0.58	0.001*,
	14	49.33±0.58	50.67±0.58	94.67±0.58	65.33±0.58	0.0001#, 0.001*,
SWIM MOTION TEST	1	43.33±0.58	45.67±0.58	22.67±0.58	21.67±0.58	0.001*,
	7	45.33±0.58	51.33±0.58	25.67±0.58	32.67±0.58	0.001*,
	14	49.33±0.58	50.67±0.58	23.67±0.58	44.33±0.58	0.0001#, 0.0001*,
PLUS MAZE TEST	1	99.67±0.58	98.67±0.58	19.33±0.58	21.33±0.58	0.001*,
	7	98	99.67±0.58	21.33±0.58	45.33±0.58	0.001*,
	14	99.67±0.58	99.33±0.58	23.33±0.58	88.33±0.58	0.0001#, 0.001*,
TURN ANGLE TEST	1	61.67±0.58	68.33±0.58	12.33±0.58	12.67±0.58	0.03*,
	7	62.33±0.58	63	16.33±0.58	42.67±0.58	0.001*,
	14	63.67±0.58	65	18.67±0.58	58.67±0.58	0.0001#, 0.001*,
COLOR PREFERENCE TEST	1	52.33±0.58	58.33±0.58	32.33±0.58	39.67±0.58	0.001*,
	7	52	56.67±0.58	36.67±0.58	41.67±0.58	0.001*,
	14	51.67±0.58	54.33±0.58	39	50.67±0.58	0.0001#, 0.001*,
N=27. G1-control group fish, G2-Vasicine group fish, G5-chemical induced group fish, G6- vasicine treated chemical induced group fish. *comparison between G5 and G2, #comparison between day1 and day 14 of G6

**Table 5:** Xenotransplantation induced neuroinflammation model

**Xeno-Transplantation Model**	**Day**	**Control**	**Vasicine**	**Xeno-transplantation**	**Xeno-transplantation + Vasicine**	**P Value**
		Mean±SD	Mean±SD	Mean±SD	Mean±SD	
Dive Tank Test	1	49.33±0.58	50.67±0.58	97.33±0.58	97.67±0.58	0.03*
	7	49.33±0.58	50.67±0.58	94.67±0.58	88.33±0.58	0.001*
	14	49.33±0.58	50.67±0.58	93	67.33±0.58	0.0001#, 0.001*
Swim Motion Test	1	43.33±0.58	45.67±0.58	18.33±0.58	17.33±0.58	0.001*,
	7	45.33±0.58	51.33±0.58	20.33±0.58	22.67±0.58	0.001*,
	14	49.33±0.58	50.67±0.58	22.67	38.67±0.58	0.0001#, 0.001*,
Plus Maze Test	1	99.67±0.58	98.33±0.58	23.33±0.58	19.67±0.58	0.001*,
	7	99.67±0.58	99	20.67±0.58	64.67±0.58	0.001*,
	14	100	100	24	73.67±0.58	0.0001#, 0.001*,
Turn Angle Test	1	61.67±0.58	68.33±0.58	22.67±0.58	26.67±0.58	0.01*,
	7	62.33±0.58	63	26	49.33±0.58	0.001*,
	14	63.67±0.58	65	28.67±0.58	58.67±0.58	0.0001#, 0.001*,
Color Preference Test	1	52.33±0.58	58.33±0.58	12.33±0.58	12.33±0.58	0.7*,
	7	52	56.67±0.58	12.67±0.58	18.67±0.58	0.001*,
	14	51.67±0.58	54.33±0.58	15.67±0.58	41.33±0.58	0.0001#, 0.001*,
N=27. Control-control group fish, Vasicine-Vasicine group fish, Xenotransplantation-xenotransplantation induced group fish, and Xenotransplantation + Vasicine- vasicine treated xenotransplantation induced group fish. *-comparison between induction model and vasicine treated fish. #-comparison between day1 and day14 of vasicine treated inflammation model.

## References

[1] Kim S (2015). Mol Cells..

[2] Münzel EJ (2014). Acta Neuropathol Commun..

[3] McGown A (2013). Ann Neurol..

[4] Simons M (2013). Curr Opin Cell Biol..

[5] Buckley CE (2010). Glia..

[6] Brösamle C (2002). Glia..

[7] Howe K (2013). Nature..

[8] Jeserich G (2008). J Mol Neurosci..

[9] Heemels MT (2016). Nature..

[10] Ghanta MK (2020). Curr Pharm Des..

[11] Ghanta M (2017). J Pharmacol Pharmacother..

[12] More SV (2013). Mediators Inflamm..

[13] Kielian T (2016). J Neurochem..

[14] Lucas SM (2006). Br J Pharmacol..

[15] Chen WW (2016). Mol Med Rep..

[16] Kempuraj D (2016). J Neurol Neurosurg Spine..

[17] Koudriavtseva T (2016). Neural Regen Res..

[18] Schulz D (2006). J Neurol..

[19] Emma Nichols (2019). The Lancet Neurology..

[20] Sasaki T (2004). J Neurochem..

[21] Balsundaram A (2018). Journal of Pharmacology and Pharmacotherapeutics..

[22] Redondo M (2012). J Med Chem..

[23] Singh B, Sharma RA (2013). Phytomedicine..

[24] Leung T (2006). Blood..

[25] Ullmann JF (2010). Neuroimage..

[26] Cooper DKC (2018). Br Med Bull..

[27] Vaz RL (2018). Front Neurol..

[28] Wallace CK (2018). J Am Assoc Lab Anim Sci..

[29] Ponzoni L (2017). Front Psychiatry..

[30] Stewart AM, Kalueff AV (2014). Brain Res..

[31] Varga ZK (2018). Sci Rep..

[32] Zhang B (2018). Chemosphere..

[33] Avdesh A (2012). J Alzheimers Dis..

[34] Ayaz Ahmed KB (2018). Sci Rep..

[35] Varela M (2012). Dev Comp Immunol..

[36] Zhang DC (2005). J Biochem Mol Biol..

[37] Gunimaladevi I (2007). Fish Shellfish Immunol..

[38] Jørgensen LVG (2018). PLoS One..

[39] Pinzón-Olejua A (2014). Neural Dev..

[40] Cheng W (2006). Dev Biol..

[41] Jin M (2018). Fish Shellfish Immunol..

[42] Ree R (2015). Biosci Rep..

[43] Diotel N (2010). J Comp Neurol..

[44] Hong YJ (2011). Ann Dermatol..

[45] Menke AL (2011). Toxicol Pathol..

[46] Erta M (2012). Int J Biol Sci..

[47] Alboni S (2010). J Neuroinflammation..

[48] Pan W (2013). Neurosci Biobehav Rev..

[49] Iughetti L (2018). Neuropeptides..

[50] Pardon MC (2016). Scientific reports..

[51] Qin XD (2012). Evid Based Complement Alternat Med..

[52] Hennige AM (2008). PLoS One..

[53] Shoaib A (2022). Cell Mol Biol (Noisy-le-grand)..

[54] Liu W (2015). PLoS One..

[55] Wakhloo RL (1980). Indian J Pharmacol..

[56] Soni S (2008). Indian J Pharm Sci..

[57] Zutshi U (1980). Planta Med..

[58] Claeson UP (2000). J Ethnopharmacol..

[59] Amla V (1987). Zhongguo Yao Li Xue Bao..

[60] Ram HN (2007). Indian Journal of Pharmaceutical Sciences..

